# Evaluation of Sample Handling Effects on Serum Vitamin E and Cholesterol Concentrations in Alpacas

**DOI:** 10.1155/2014/537213

**Published:** 2014-01-12

**Authors:** Andrea S. Lear, Stacey R. Byers, Robert J. Callan, Jessica A. A. McArt

**Affiliations:** Department of Clinical Sciences, College of Veterinary Medicine and Biomedical Sciences, Colorado State University, Fort Collins, CO 80523-1620, USA

## Abstract

Clinical cases of vitamin E deficiencies have been diagnosed in camelids and may indicate that these species are more sensitive to inadequate vitamin E in hay-based diets compared to other ruminant and equine species. In bovine, cholesterol has been reported to affect vitamin E concentrations. In order to evaluate vitamin E deficiencies in camelids, the effects of collection and storage of the blood samples prior to processing were necessary. Reports vary as to factors affecting vitamin E and cholesterol in blood samples, and diagnostic laboratories vary in instructions regarding sample handling. Blood was collected from healthy alpacas and processed under conditions including exposure to fluorescent light, serum and red blood cell contact, tube stopper contact, temperature, and hemolysis. Serum vitamin E and cholesterol concentrations were then measured. Statistical analyses found that the vitamin E concentrations decreased with prolonged contact with the tube stopper and with increasing hemolysis. Vitamin E concentration variations were seen with other factors but were not significant. Time prior to serum separation and individual animal variation was found to alter cholesterol concentrations within the sample, yet this finding was clinically unremarkable. No correlation was seen between vitamin E and cholesterol concentration, possibly due to lack of variation of cholesterol.

## 1. Introduction

Vitamin E is an important nutrient with many critical antioxidant functions throughout the body. This vitamin is found in cellular membranes as well as intracellular and extracellular fluid. Deficiencies can manifest in a wide variety of medical conditions [1]. The muscular and neurological systems are affected most commonly; however, conditions affecting the immune and reproductive systems are reported [2, 3]. Mammals do not synthesize vitamin E, and the most abundant sources for herbivores are fresh green forages [4, 5]. Once forage is cut, dried, and packaged as hay, the vitamin E concentration rapidly decreases within days below nutritional requirements and animals fed a hay diet require additional supplementation [4–6].

Clinical and subclinical vitamin E deficiencies can be diagnosed in animals in any geographical location with limited access to fresh forage, as has been seen in many locations in the United States due to significant drought conditions. Deficiencies can also be exacerbated by increased cellular demands due to metabolic oxidative stresses and most often manifest as myopathies (e.g., nutritional myopathy and nutritional muscular dystrophy) and neuropathies (e.g., diaphragmatic paralysis and equine motor neuron disease) [7–9]. Ill-thrift and reproductive problems have also been documented [10, 11]. Camelids in many areas of the United States are raised on dry-lot operations and fed a strictly hay-based diet. Cases of diaphragmatic paralysis and consistently low vitamin E concentrations have been observed in these animals versus those raised on pasture-based operations ([9] and personal communication).

Vitamin E studies in humans and some veterinary species have identified sample handling factors that have the potential for altering serum vitamin E concentrations. These factors include contact with the blood tube stopper, time and temperature before separation of the serum from the red blood cells, and hemolysis of the blood sample [12–16]. Additionally due to the lipid soluble nature of vitamin E, the ratio of vitamin E to cholesterol is now evaluated by some reference laboratories [17–19]. Human and bovine cholesterol concentrations have been described as stable under these storage parameters [20, 21].

The proper collection and storage methods to minimize the effects on vitamin E and cholesterol concentrations have not been verified for camelid serum samples. Blood collection from camelids can be a challenging procedure and hemolysis may be more common than in other species. In field collection situations, tubes are often not spun down immediately and may be kept warmer than recommend by diagnostic laboratories, and blood often contacts the rubber tube stopper. Camelid RBCs are smaller and elliptical in shape compared to other mammalian RBCs, and the effects of the different physiological features of the RBCs and potential effects on vitamin E and cholesterol need to be verified. Additionally, despite research showing that ultraviolet light does not affect vitamin E levels, some laboratories still request samples to be stored and shipped without light exposure.

The purpose of this study was to determine how sample-handling conditions of temperature, time, contact with the tube stopper, and exposure to light affect serum vitamin E and cholesterol concentrations. The effect of hemolysis on serum vitamin E and cholesterol concentrations was also studied.

## 2. Materials and Methods

### 2.1. Animals

This study was approved by the Colorado State University (CSU) Institutional Animal Use and Care Committee. Two apparently healthy male castrated adult alpacas (approximately 2 years old) were used for sample collection. The animals were housed in an outdoor pen and fed grass hay without further vitamin or mineral supplementation. For blood collection, the animals were restrained in an alpaca chute. The cranioventral aspect of the neck was clipped to remove fiber from a 6 square cm area of skin over the right jugular vein. The skin was aseptically prepared and a 14 gauge, 7.62 cm intravenous catheter (Mila International, Inc. 12 Price Avenue, Erlanger, KY, USA) was placed. Blood was collected in 12 mL syringes directly from the catheter. Approximately 220 mL of blood was collected from each animal and immediately divided evenly into 54–4 mL nonanticoagulant (red top) blood collection tubes (Becton Dickinson and Company, Inc. Sandy, UT, USA).

### 2.2. Sample Handling

The effect of exposure to fluorescent room light, contact with the red rubber tube stopper, duration (1, 4, and 24 hours) of storage, and temperature (room temperature (25°C) or refrigeration (4°C)) until serum separation was tested using a partial factorial design. The effect of light was not evaluated for the samples placed under refrigeration. All processing combinations were performed in triplicate for each animal. Following treatment, serum was collected from the tubes following centrifugation at 1,000 ×g for 10 minutes. Serum was then transferred to 2 mL freezer vials and stored at −20°C until vitamin E and cholesterol analyses were performed. Vitamin E concentrations were determined using high-pressure liquid chromatography (Waters 474 Scanning Fluorescence Detector, Waters Corporation, 34 Maple Street, Milford, MA, USA) at the CSU Veterinary Diagnostic Laboratory. This analyzer was calibrated using manufacturers recommendations and performed between each batch of samples evaluated for vitamin E concentrations. Cholesterol concentrations were determined at the CSU Clinical Pathology Laboratory using a Hitachi 917 chemistry analyzer (Hitachi High Technologies America, Corporation. Wallingford, CT, USA) as directed by the manufacturer guidelines.

The effects of hemolysis on serum vitamin E and cholesterol concentration were evaluated by the addition of RBCs to serum samples and then subjected to 3 freeze-thaw cycles as described by Hooser et al. [13]. Forty milliliters of whole blood was collected from each alpaca and divided between 1–4 mL EDTA tube and 9–4 mL red top serum tubes. The RBC concentration of the whole blood was determined by microscopic evaluation on a hemocytometer. The serum tubes were allowed to clot for 1 hour at room temperature and serum was collected as described above. Serum was separated and divided into 2 mL aliquots for each alpaca. Red blood cells from the whole blood sample were added to the aliquots of serum to prepare concentrations of approximately 0 (negative control), 1 × 10^6^, 1 × 10^7^, 1 × 10^8^, and 1 × 10^9^ RBC/mL. The CSU Clinical Pathology Laboratory reference RBC count in camelids is approximately 11.3 to 17.6 × 10^9^ RBC/mL. This protocol resulted in a relative percent hemolysis ranging from about 0.01 to 10%. Samples were prepared in triplicate for each animal. Tubes were stored upright and frozen at −20°C for 24 hrs and thawed at room temperature (25°C) for 3 cycles. Samples were then centrifuged as described above and the serum separated to remove any remaining RBC fragments and stored at −20°C until processing. Serum samples were submitted to the CSU Clinical Pathology Laboratory to determine a quantitative hemolysis score using a Hitachi 917 chemistry analyzer as directed by the manufacturer guidelines. Vitamin E and cholesterol concentrations were also determined for these samples as described above.

### 2.3. Statistical Analysis

The effects of individual alpaca, exposure to fluorescent room light, contact with the rubber tube stopper, storage duration before serum separation, and temperature during storage on serum vitamin E concentration were analyzed through an ANOVA using the GLM procedure of SAS (SAS Institute, Inc., Cary, NC, USA) A *P-*value < 0.05 was considered significant. The relationship of serum vitamin E and cholesterol with hemolysis in induced samples was evaluated by linear regression using the CORR procedure of SAS.

## 3. Results and Discussion

**Figure 1:**
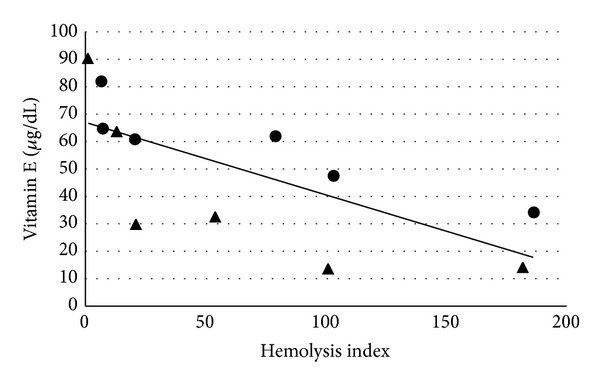
Least squares means serum vitamin E concentrations (*μ*g/dL) versus induced serum hemolysis index in 2 adult male alpacas. Alpaca A is represented by the solid circle and alpaca B by the solid triangle. A best-fit line, corresponding to correlation coefficient of −0.69, is present.

Mean vitamin E concentrations for each sample processing technique from each alpaca are presented in Table 1. The average SD for all triplicate samples was 5.4 *μ*g/dL. Vitamin E concentration was found to be significantly different due to tube position; no other processing condition had an effect on vitamin E concentration.  Figure 1 illustrates a strong negative correlation (*r* = −0.69) observed between induced hemolysis index and vitamin E (*P* < 0.0001). Cholesterol concentrations for each sample processing technique from each alpaca are presented in Table 2. Cholesterol concentration was found to significantly vary between the individual animals and time prior to serum separation. A weak negative correlation (*r* = −0.33) was seen between cholesterol concentration and induced hemolysis index (*P* = 0.04) (figure not included).

Contact with the red rubber stopper decreased vitamin E concentrations, which may lead to a false interpretation of vitamin E deficiency. The cause of this change is unknown but may be due to a reaction with the red rubber top and subsequent utilization or oxidation to an unmeasureable form of vitamin E [12]. No other sample processing condition evaluated in this study had a significant effect on variation in vitamin E concentration; thus maintaining the sample tube in an upright position prior to analysis is important in obtaining the most accurate serum vitamin E concentration. Even though fluorescent light was not found to significantly affect vitamin E concentrations, the effects of ultraviolet light were not evaluated, therefore limiting exposure to UV light is recommended as clinical implications cannot be hypothesized from this study.

Vitamin E concentrations were performed in triplicate using high-pressure liquid chromatography. A wide variability in measured vitamin E concentrations was seen between the triplicate evaluations for the same serum sample. High-pressure liquid chromatography detects vitamin E in the DL-alpha tocopherol form, which can be easily denatured during preparation and analysis leading to inconsistency between concentrations of the same serum sample. In addition, a small volume of serum was submitted for each test in order to perform this test in triplicate, resulting in possible unevenness within the sample vitamin E concentrations. Other possible means of variability introduction included the multistep process of preparing the sample for analysis as well as individual laboratory technician differences in preparation of the samples.

In this experiment, blood samples were collected via jugular catheters to reduce hemolysis in the original samples. In the artificially induced hemolysis samples, only moderate to marked gross hemolysis index was found to decrease vitamin E concentrations in alpaca serum, in agreement with studies from other species [13]. Therefore hemolysis should be minimized when possible during collection and handling of samples prior to vitamin E analyses to ensure the most accurate results. Time prior to serum separation was found to effect cholesterol concentrations, yet this finding was not clinically relevant due to the small numerical difference in mean cholesterol concentrations between time outcomes. While a weak negative correlation was observed between induced hemolysis index and cholesterol concentrations, in general, cholesterol was found to be very stable across sample-handling parameters. Interestingly, a large variation in cholesterol concentration was observed between individual animals. In other species falsely elevated levels of serum vitamin E are observed during times of high fat mobilization due to a positive correlation between vitamin E and cholesterol concentrations [17, 18, 21–23]. Conversely, falsely low levels are seen during times of low fat mobilization. If camelids do experience cholesterol fluctuations, this may be an important factor for accurate identification of vitamin E status in camelids. Further data is needed to fully evaluate this relationship.

## 4. Conclusions

These results indicate that decreasing hemolysis during sample collection and upright storage prior to analysis will provide a more accurate vitamin E evaluation in alpacas. Although cholesterol variations were seen with certain handling parameters, none were clinically relevant. Additional research regarding vitamin E in alpacas is needed to better evaluate the effects of dietary deficiencies and supplementations required.

## Conflict of Interests

The authors declare that there is no conflict of interests regarding the publication of this paper.

## Figures and Tables

**Table 1:** Least squares means serum vitamin E concentrations (*µ*g/dL) in 2 adult male alpacas after exposure of whole blood to the following conditions: exposure to fluorescent room light (light exposure: light or dark), contact with the rubber tube stopper (position: upright or stopper contact), storage duration before serum separation (time: 1, 4, or 24 hours), and temperature during storage (temp.: 4°C or 25°C). The effect of each alpaca was also analyzed. *P* values are reported for the ANOVA *F* statistic of the entire variable.

Variable	Mean (SE)	*P* value
Alpaca		
A	44.3 (3.0)	0.25
B	48.5 (3.0)	
Light exposure		
Light	48.4 (4.0)	0.38
Dark	44.3 (2.3)	
Position		
Upright	57.5 (3.0)	<0.0001
Stopper contact	35.2 (3.0)	
Time		
1 hr	44.7 (3.5)	0.05
4 hr	52.8 (3.5)	
24 hr	41.6 (3.5)	
Temp.		
4°C	46.4 (4.0)	0.99
25°C	46.3 (2.3)	

**Table 2:** Least squares means serum cholesterol concentrations (*µ*g/dL) in 2 adult male alpacas after exposure of whole blood to the following conditions: exposure to fluorescent room light (light exposure: light or dark), contact with the rubber tube stopper (position: upright or stopper contact), storage duration before serum separation (time: 1, 4, or 24 hours), and temperature during storage (temp.: 4°C or 25°C). The effect of each alpaca was also analyzed. *P* values are reported for the ANOVA *F* statistic of the entire variable.

Variable	Mean (SE)	*P* value
Alpaca		
A	60.7 (0.21)	<0.0001
B	42.9 (0.21)	
Light exposure		
Light	51.8 (0.28)	0.99
Dark	51.8 (0.17)	
Position		
Upright	51.7 (0.21)	0.54
Stopper contact	51.9 (0.21)	
Time		
1 hr	51.0 (0.25)	0.0004
4 hr	52.1 (0.25)	
24 hr	52.3 (0.25)	
Temp.		
4°C	52.1 (0.28)	0.08
25°C	51.5 (0.16)	
